# Supplementary material for: Learning auditory discriminations from observation is efficient but less robust than learning from experience
Source: Nat Commun. 2018 Aug 13;9:3218. doi: 10.1038/s41467-018-05422-y (PMC6089935; doi:10.1038/s41467-018-05422-y)
Supplement: Supplementary file 1 — Supplementary Information [file 41467_2018_5422_MOESM1_ESM.docx]

**SUPPLEMENTARY INFORMATION**

Title: Learning auditory discriminations from observation is efficient but less robust than learning from experience

Narula, Herbst, Rychen, Hahnloser 2018.

**Supplementary figures**

**
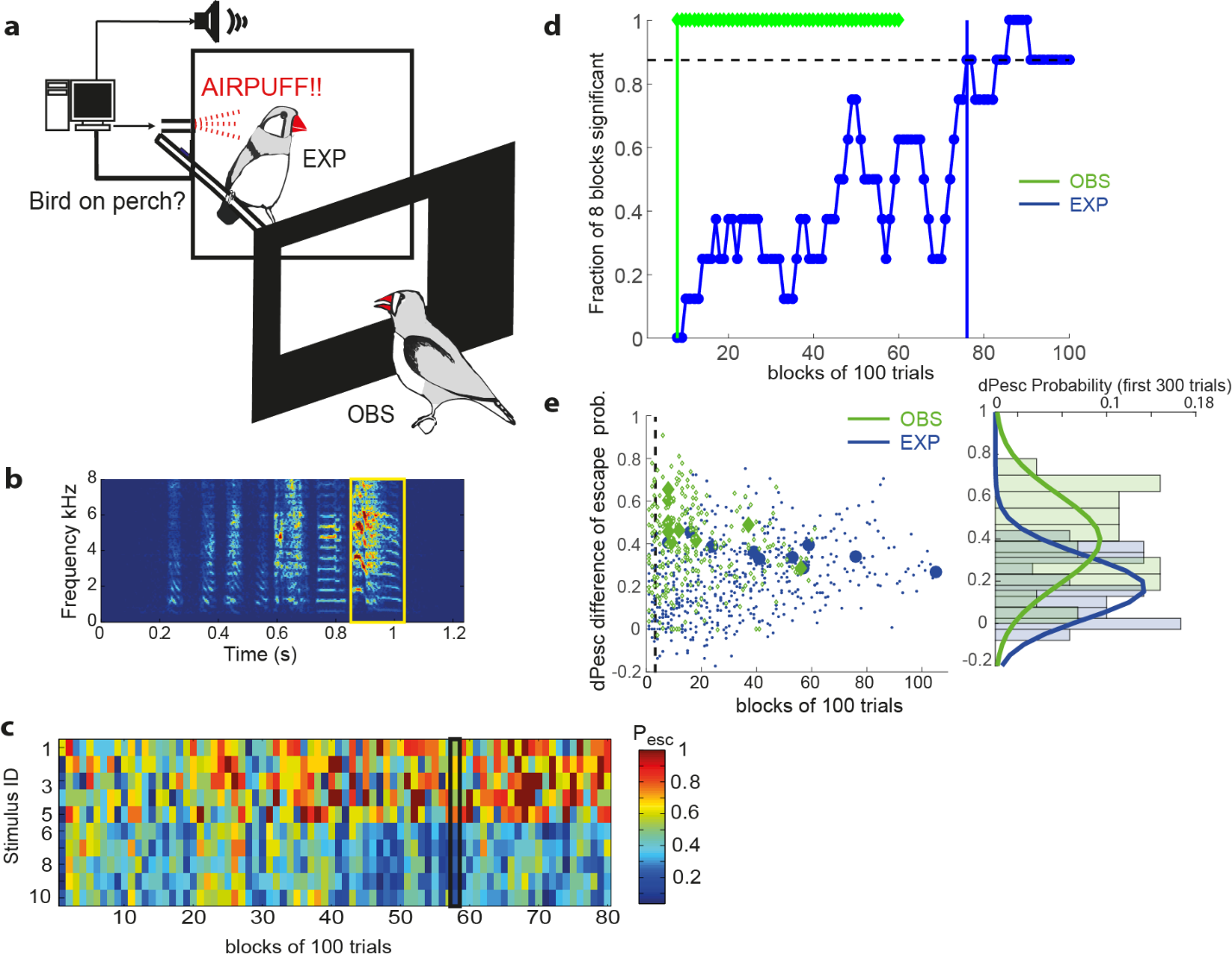
**

**Supplementary Figure 1.** **Experimental setup, stimulus, and** **dynamics of discrimination learning** (**a**) An experimenter (EXP) and observer (OBS) zebra finch pair were housed in adjacent cages separated by a screen that restricted visual interactions to an experimenter perch equipped with an air-puff delivery mechanism. (**b**) Log power spectrogram of a zebra finch song. The stimuli were composed of different renditions of a complex song syllable (yellow rectangle). (**c**) Heat map showing the probability of escape (P_esc_) as a function of stimulus ID (same bird as in Fig 2b). For this bird, the first five stimuli (‘short’ class) were punished with an air-puff. There is no clear difference between the rates of increase or decrease of P_esc_ for stimuli that are furthest from the decision boundary (S_1_ and S_10_) compared to stimuli straddling the boundary (S_5_ and S_6_). The criterion block is indicated with a black rectangle. These dynamics are typical for EXP. (**d**) Criterion dynamics in an experimenter-observer pair: the observer reaches the statistical criterion (vertical green line) after a minimum of 8 blocks, but the experimenter needs 76 blocks (vertical blue line) (same birds as in Fig 2c). Criterion is defined as 7/8 statistically significant blocks of 100 trials each (horizontal dashed line). A block is “significant” if the p-value of the z-test of individual proportions on dPesc is < 0.01. (**e, left**) Scatter plot of dPesc as a function of trial block number (n=10 EXP, blue dots; n=9 OBS, green diamonds). dPesc at the criterion is depicted with a larger, solid symbol. (**e, right**) Probability distributions of dPesc for EXP (blue, n=10) and OBS (green, n=9) in the first 3 blocks (300 trials, up to dashed line), fitted with Gaussian functions. Panel a was drawn by Gagan Narula.


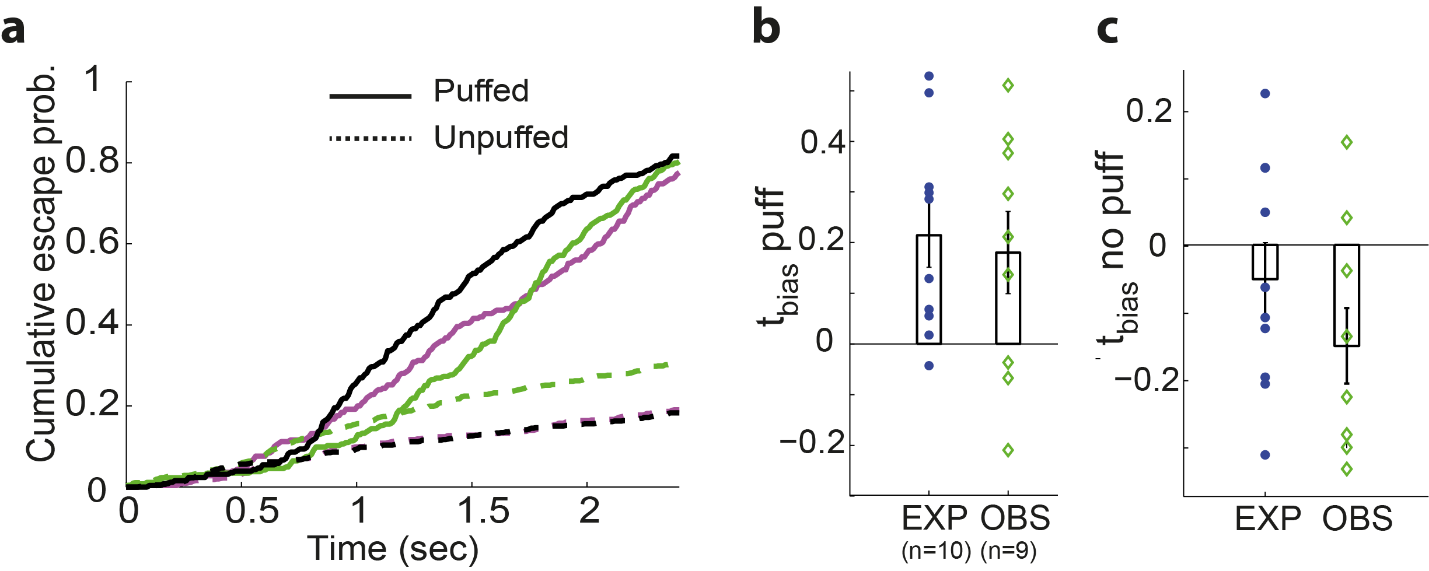


**Supplementary Figure 2.** **Within-trial analysis shows sudden increase in escape rates in both OBS and EXP.** (**a**) Cumulative escape probabilities as a function of trial time (puffed trials indicated by solid line and unpuffed trials by dashed line), shown for three OBS after they reached the criterion. All three birds tend to avoid air puffs by increasing the escape rate (increased slope in curves) within 1 s of trial onset. (**b** and **c**) Escape time bias for puffed trials and unpuffed trials, respectively. On average, in puffed trials, EXP and OBS escaped after the mid-trial time point (mean bias in EXP = 0.21 s, OBS = 0.18 s) and in unpuffed trials they escaped slightly before that point (mean bias EXP = -0.04 s, OBS = -0.15 s).


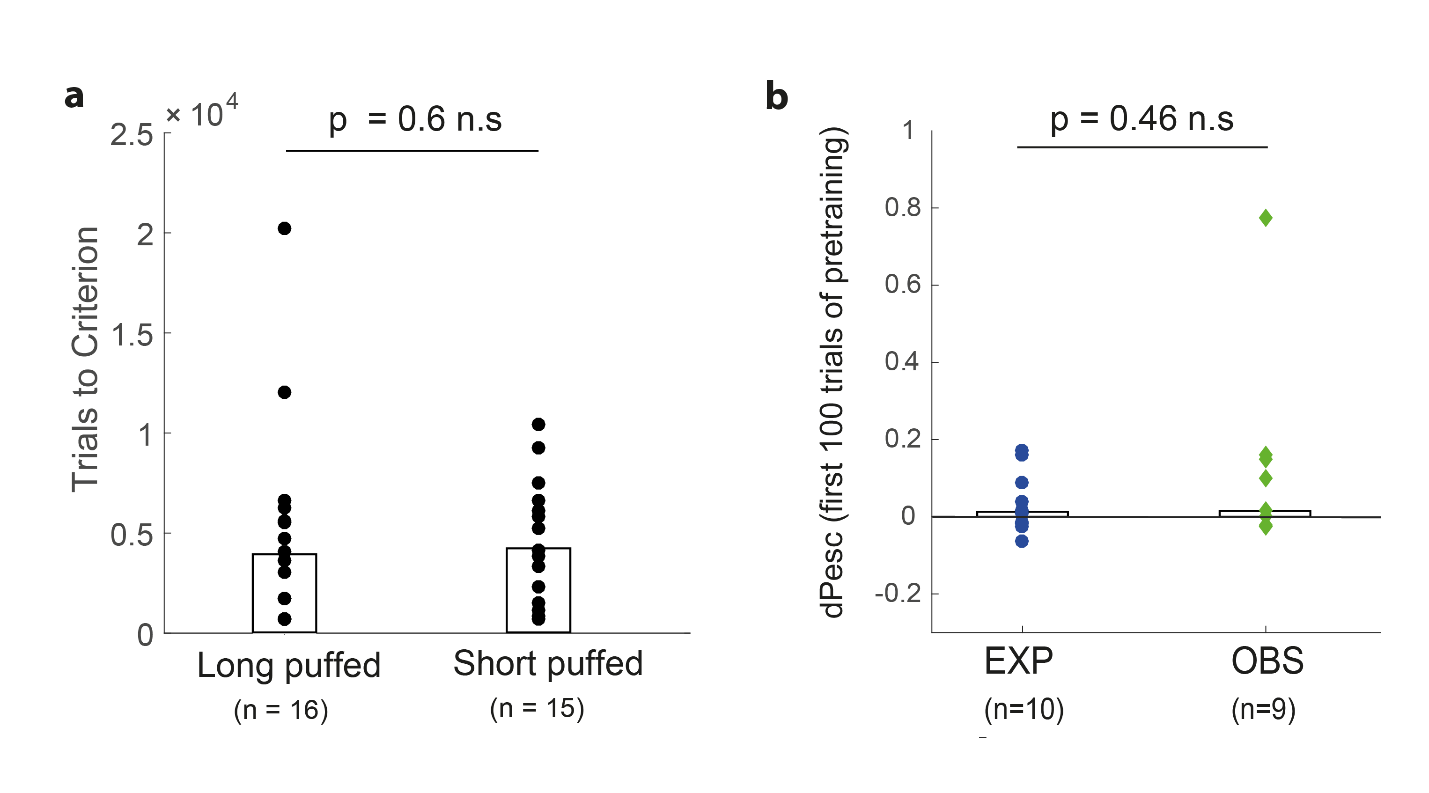


**Supplementary Figure 3. Performance comparison between counterbalanced air-puff conditions, and control against stimulus enhancement.** (**a**) No difference in the trials to criterion between birds trained with long stimuli puffed versus short stimuli puffed (generalization set not included). Long puffed (n=16): 3900 [800, 20300] (median [range]). Short puffed (n=15): 4200 [800, 10500]. No significant difference in group medians (p = 0.6, test statistic = 106.5, Wilcoxon rank sum test). (**b**) Neither EXP nor OBS showed a significantly higher escape rate for puffed than unpuffed stimuli (compared to 0) during the first block of the pre-training and pre-test phases, respectively: EXP dPesc: 0.013 [-0.06, 0.17]; null hypothesis of EXP dPesc ≠ 0 not rejected, p = 0.37, test statistic = 37, Wilcoxon sign rank test. OBS dPesc: 0.015 [-0.36, 0.77]; null hypothesis of OBS dPesc ≠ 0 not rejected, p = 0.44, test statistic = 24, Wilcoxon sign rank test. Observer and experimenter performance was very similar (null hypotheses of EXP dPesc = OBS dPesc not rejected, p = 0.46, test statistic = 43.5, Wilcoxon rank sum test).

**
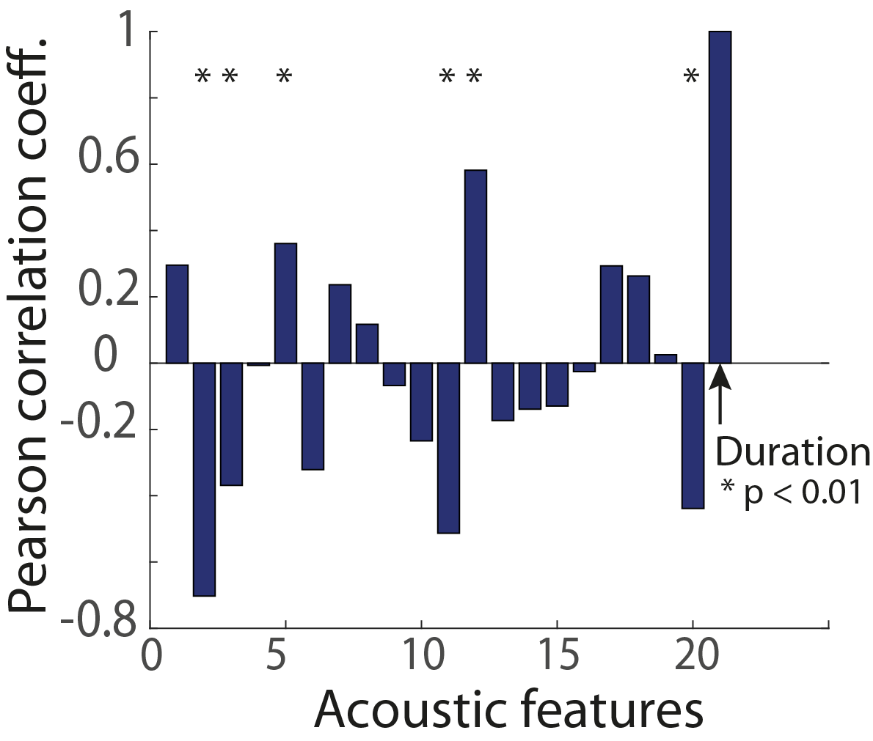
**

**Supplementary Figure 4. Pearson correlation coefficient between duration and other acoustic features** (such as frequency modulation, mean pitch, pitch goodness, amplitude modulation etc.) computed over the 60 syllables in the training set (5 stimuli x 6 syllables per stimuli x 2 classes). The * indicates statistical significance with p<0.01.


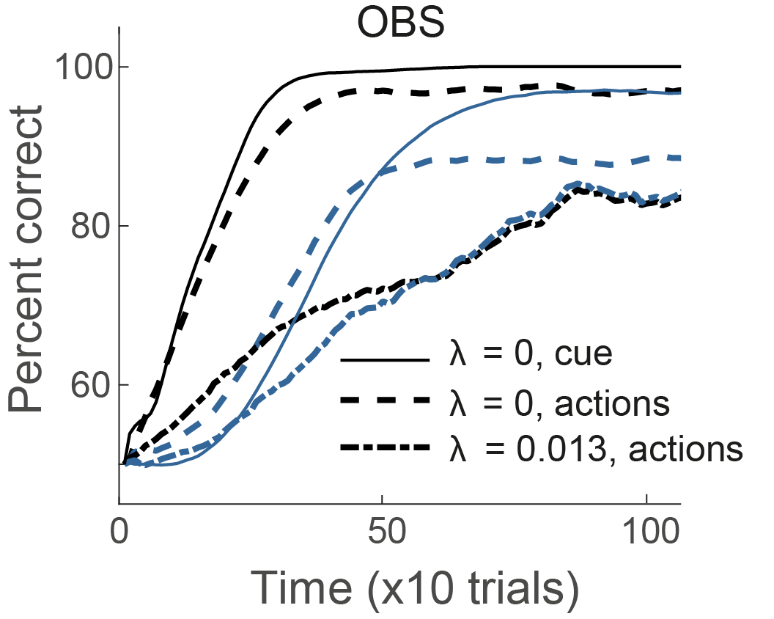


**Supplementary Figure 5. Effects of learning under noisy experimenter actions.** Learning curves in ‘observer’ neurons are plausible both when OBS learn from the auditory cues of air-puffs (full lines) or from experimenter actions (cues affected by 30% random ‘label noise’, dashed lines). However, training and generalization performances get too close to be realistic when observers learn with L1-regularization and 30% label noise (dash-dotted line).

Supplementary Tables

**Supplementary Table 1. List of features computed by Sound Analysis Pro (SAP 2011) software for our stimuli from the Training set (60 syllables, 21 acoustic features).**

| Feature number | FEATURE NAME | FEATURE DESCRIPTION | PEARSON CORRELATION WITH DURATION (and p-value, * = significant at 99% level) |
| --- | --- | --- | --- |
| 1 | Mean amplitude modulation | Average (across a syllable) time derivative of power (summed) over all frequencies within a given band | 0.295 (0.03) |
| 2 | Variance of amplitude modulation | Variance (across syllable) of amplitude modulation | -0.73 (3.85*10^-10^***) |
| 3 | Mean frequency modulation | Average (across syllable) slope of the derivative of power over time and frequency | -0.37 (0.0037 **) |
| 4 | Variance of frequency modulation | Variance (across syllable) of frequency modulation | -0.007 (0.96) |
| 5 | Mean Wiener Entropy | Average (across syllable) of the width and uniformity of power spectrum. Noisy, broadband signals have value near 1, and pure tones are near 0. | 0.361 (0.005 **) |
| 6 | Variance of Wiener entropy | Variance (across syllable) of Wiener entropy | -0.32 (0.012) |
| 7 | Mean amplitude | Average (across syllable) amplitude of the signal (log-scale summed Power over all frequencies) | 0.24 (0.07) |
| 8 | Variance of amplitude | Variance (across syllable) of amplitude | 0.12 (0.37) |
| 9 | Mean Gravity center | Average (across syllable) of the center of the distribution of power across frequencies | -0.07 (0.61) |
| 10 | Variance of Gravity center | Variance (across syllable) of gravity center | -0.23 (0.07) |
| 11 | Mean Pitch goodness | Average (across syllable) of the amount of periodicity in the sound. Harmonic sounds have high pitch goodness, whereas noisy sounds and pure tones have low values. | -0.51 (2.79*10^-5^ ***) |
| 12 | Variance of Pitch goodness | Variance (across syllable) of pitch goodness | 0.58 (1.11*10^-6^ ***) |
| 13 | Mean Pitch | Average (across syllable) of the fundamental frequency (from YIN algorithm) | -0.17 (0.18) |
| 14 | Variance of pitch | Variance (across syllable) of fundamental frequency (from YIN algorithm) | -0.14 (0.29) |
| 15 | Mean Peak frequency | Average (across syllable) of the frequency of maximum power in the spectrum | -0.13 (0.32) |
| 16 | Variance of Peak frequency | Variance (across syllable) of the peak frequency | -0.025 (0.85) |
| 17 | Mean Pitch weight | Average (across syllable) of the peak frequency weighted by the normalized mean pitch goodness (amount of periodicity in harmonics see pt 11). | 0.29 (0.023) |
| 18 | Variance of Pitch  Weight | Variance (across syllable) of pitch weight | 0.26 (0.043) |
| 19 | Median Pitch in specific time window 1 | Median pitch computed using the harmonic power spectrum method in three 16 ms wide windows: 20 ms, 24 ms, and 28 ms after the onset of the syllable | 0.025 (0.85) |
| 20 | Median Pitch in specific time window 2 | Median pitch computed using the harmonic power spectrum method in three 16 ms wide windows: 32 ms, 36 ms, and 40 ms after the onset of the syllable. | -0.44 (0.0005 ***) |
| 21 | Duration (s) | Duration of the syllable in seconds | 1.0 (not applicable) |

Supplementary Note 1

**Experimental animals**

We used adult (older than 90 days post hatch, dph) female zebra finches (*Taeniopygia guttata,* N = 51 females) raised in our colony. Zebra finches are useful models for studies of sensory learning thanks to their ability to detect subtle differences among natural stimuli such as complex contact calls^1–3^ and highly stereotyped songs^4,5^. Within these stimuli, they can accurately detect changes in the fine temporal structure occurring within 1.2 ms^6^. They are capable of temporal integration of pure tones^7^ and are even sensitive to variations in human speech^8,9^. Their learning in stimulus playback experiments is dependent on cues such as behavioral context^10,11^, which makes them suitable for our comparative study of discrimination learning. We chose to study observation learning in females for two reasons. First, females choose their mates by listening to the songs of competing males. Therefore, it is natural to assume that they attend to the behavior of other birds. Second, the use of female zebra finches allows for greater flexibility in planning the breeding cycles and logistics of a zebra finch colony maintained for experiments on males, the predominantly used sex. All experiments were licensed by the Veterinary Office of the Kanton of Zurich.

**Experimental setup**

We adapted an operant conditioning paradigm using social reinforcement^12,13^. During the experiment, all birds were housed with unrestricted access to food, water, grit, and cuttle bone in individual cages with dimensions 30 x 30 x 40 cm, placed inside a custom sound isolation chamber. The chamber contained a speaker for playing the stimuli, a microphone for sound recordings, and a webcam.

The cages for experimenters and observers were placed adjacent to each other. Each cage contained three perches, two perches for food and water access and a third (window) perch for looking into the other cage. We placed a cardboard screen with a small (15 x 15 cm) peeping window between the two cages to block the sight into the other cage from all vantage points other than the window perch, Supplementary Figure 1a. Experimenters and observers frequently visited their window perches (henceforth referred to as “perches”) to interact with each other. Any action performed by the perching experimenter, such as flying away, was clearly visible to the perching observer.

The perch of the experimenter contained a magnet and Hall sensor to detect upward (and downward) movements of the perch, which we used to read out the presence (or absence) of the animal, Supplementary Figure 1a. The observer’s perch was not equipped with a Hall sensor.

While the experimenter perched, we played stimuli through the loudspeaker. As an aversive reinforcement agent, we delivered puffs of air to the bird in a direction parallel to the perch. Air-puffs were delivered through an outlet of 1 cm diameter and at a pressure level of 7 bar. The effect of the puff was to displace the bird from the perch and to disrupt its social experience. Because social interaction and air-puffs are strong motivational and reinforcing agents respectively, we obtained hundreds of stimulus-response trials each day. None of the birds were injured by air-puffs. However, the air-puffs were quite loud (max 107 dB at experimenter perch and 104 dB at observer perch).

**Stimuli**

*Duration discrimination*

We created a set of 10 stimuli synthesized from the songs of an adult male zebra finch (o7r14) from our colony. Songs were filtered with a 4^th^ order Butterworth high-pass filter (600 Hz cut-off) and digitized at 32 kHz. We collected all renditions of a particular syllable produced during one day of singing. We computed syllable durations via thresholding of sound amplitude traces. Based on the full range of produced syllable durations we defined ten stimuli of increasing duration. Each stimulus S_i_ in this set (i=1,2, …,10) was made of a string of six syllable renditions, wherein each rendition was longer than the six renditions in stimulus S_i-1_, Fig. 1b. Within a stimulus, the six renditions were arranged in order of increasing duration. In total, the stimulus set comprised sixty different syllable renditions (10 stimuli of 6 renditions each). To avoid sound onset artifacts, we smoothed syllable onsets and offsets by multiplying the sound waveform in the time domain with sigmoid functions of width σ=16 ms. Inter-syllable gaps were 22 ms.

Based on the ten stimuli we defined two stimulus classes: the class ‘short’ was formed by stimuli S_1_ to S_5_, and the class ‘long’ was formed by stimuli S_6_ to S_10_, Fig 1c. The stimuli were distinguishable based on duration but also based on other sound features, allowing birds to ‘overfit’ their discriminative systems.

We implemented a Go-NoGo operant conditioning paradigm using aversive air puffs that followed stimuli from one of the two classes. We counterbalanced the aversively reinforced class (short or long) across birds (24/54 birds were puffed after short stimuli). In n = 31 birds exposed to the training set, there was no difference in learning time between short- or long-puffed birds, Supplementary Figure 3a. In the main text and in the following, we use the terms ’puffed’ and ‘unpuffed’ as class labels, irrespective of whether short or long stimuli were reinforced.

We refer to the stimulus set {S_1_ , …, S_10_} as the *training set*. To create a *generalization set,* we formed another set of 10 stimuli {S’_1_, …,S’_10_} from renditions of the same syllable recorded on the very next day, Fig. 1b right. The stimulus durations in this *generalization set* tended to be slightly shorter than in the training set, including near the class boundary, Fig. 1c.

**Controlling vocal communication between experimenters and observers**

To implement dynamic control of vocal communication (in the main text, these OBS were referred to as -TCOM, n = 5 birds), we separated EXP and OBS into acoustic isolation boxes with windows that allowed for visual interactions. Echo cancellation was implemented on a field programmable gate array (FPGA), which garantees a low latency (<1 ms) and flexible signal processing. We developed the sytem on a Compact-RIO (Reconfigurable Input and Output) cRIO-9063 with an Atrix-7 FPGA and a realtime (RT) Linux Controller, all from National Instruments Inc. The system was connected over a standard internet connection to a host computer, on which a graphical user interface allowed monitoring the signals and controling various experimental parameters. The signal processing software on the FPGA as well as the graphical user interface running on a standard windows PC were developed with LabVIEW from National Intruments.

Data were acquired with a recorder system also written in LabView. This system acquired perching signals and sounds and it produced acoustic stimuli and triggered air-puff trigger signals. The recorder system also controlled the communication system to block communication between EXP and -TCOM during trial times (from stimulus onset to air-puff offset).

We prevented transmission of feedback-related echoes from one box to the other using an echo cancellation system. In this system, the microphone signal was amplified and digitized (National Instruments cRIO NI-9215, 96 kS/s, 16 bits, ±10 V range) and fed into the FPGA. All input signals were bandpass filtered from 500 Hz – 8 kHz (Butterworth FIR filter of order 16 with stopband attenuation of 20 dB at 350 Hz and 10 kHz). The echo cancellation was implemented as a Least Mean Square (LMS) filter (with 256 filter coefficients) that optimally modelled the microphone response to white noise played through the loudspeaker. The LMS-filtered speaker signal thus formed an estimate of the “echo” produced by the box. By subtracting the LMS-filtered speaker signal from the microphone signal, we achieved an echo suppression of roughly -22 dB (on white noise). During trial times, we completely suppressed signal transmission between the boxes.

**Bird groups and experiment hypothesis (detailed information):**

We used six different groups of experimenters and observers, as follows:

1. Experimenters (**EXP**, n=10 birds): These birds were trained to escape from the perch prior to arrival of air-puffs. The birds first underwent a pre-training phase in which they were accustomed to the setup, followed by a training phase (see *Procedure*). Three out of nine experimenters were also tested on a generalization set of stimuli (Generalization phase) once the training phase was completed, Fig. 2a left. Each phase ended when the bird’s performance reached a set criterion (see *Performance measures and Statistical Criterion*).
2. Observers (**OBS**, n = 9 birds): Observers were subjected to three phases: an observation phase in which they observed the entire pre-training and training phases of an experimenter, a pre-testing phase (identical to the experimenter’s pre-training phase), and a testing phase (identical to the experimenter’s training phase), Fig. 2a right. In the testing phase, OBS were tested on the training set {S_1_,…,S_10_}.
3. Generalizing experimenters (**GENEXP**, n = 9 birds): these birds were tested on the generalization set of stimuli after they had finished the pre-training and training phases on the training set, Fig. 3a top and bottom left.
4. Generalizing observers (**GENOBS**, n = 9 birds): These birds underwent an observation phase in which they could observe experimenters’ reactions to the training set. This observation phase was followed by a pre-testing phase which was identical to the pre-testing phase of OBS. Thereafter, during the testing phase, GENOBS were tested on the full generalization set, Fig. 3a bottom right.
5. Perceptual learners (**PLs**, n = 7 birds): We tested whether observers could perceptually learn the relationship between individual acoustic stimuli and the presence or absence of a loud air-puff sound (Fig. 2b, spectrogram, red arrows) that constitutes a label of stimulus class (puffed/unpuffed). If observers learned this relationship while ignoring the experimenter’s behavior, they would only need to learn the aversive value of the air-puff during testing (or more likely during pre-testing) to trigger an appropriate escape response. Most importantly, the puff-escape association may be acquired much faster than the stimulus-puff association learned perceptually. To test for this simple perceptual learning effect, we replaced the observation phase in OBS with a perceptual learning phase in PL. Initially, PLs were allowed to watch an experimenter trigger several thousand stimuli and air-puffs. However, in their case, the air-puffs were directed away from the experimenter (oriented downwards outside the cage) so PLs never experienced or saw the effect of an air-puff against a bird prior to entering the pre-testing phase. After completion of the pre-testing phase, PLs were subjected to the testing phase, Fig. 4a.
6. Valence learners (**VLs**, n = 5 birds): After finding fast learning in OBS and slow learning in PLs, we wondered whether the observers’ learning benefits required an expert model. We wanted to control for whether the mere presence of a naively behaving experimenter, which shows no sign of stimulus discrimination but is frequently struck by air-puffs, may induce stimulus-reward association learning in the observer through a Rescorla-Wagner^14^ type of learning rule. This form of social learning can also be referred to as “observational conditioning”, postulating that observers learn about the outcome (the valence) of an experimenters’ actions given stimuli, rather than the actions themselves^15,16^. To test against observational conditioning, we allowed n=5 VLs to observe naive experimenters prior to their pre-testing phase. In addition, n=3 isolated VLs were given several hundred stimulus-puff pairings (same protocol as training phase in EXP) prior to observing a naive experimenter. After completion of the pre-testing phase, VLs were subjected to the testing phase, Fig. 4b.
7. No Trial Communication learners (**-TCOMs**, n = 5 birds): To test against learning in observers from vocal cues (or the lack thereof, Fig. 4 c,d) we separated five observers from their experimenters (pre-training and training phases) into an adjacent, acoustically isolated box. These observers (-TCOMs) could view the EXP through a window and communicate with them vocally through a custom (software controlled) communication channel except during trial periods defined from stimulus onset to air-puff offset. During trial periods, -TCOMs could only hear the stimuli and the sounds of air-puffs, but no sounds triggered by the experimenter. After completion of the EXP training phase, -TCOMs were subjected to the pre-testing and testing phases as for OBS.

**Procedure**

*Pre-training phase in experimenters*

In experimenters, an experiment began with a *pre-training phase* that lasted three to four days. In this phase, we accustomed experimenters to the events occurring in the task, which are the strings of six syllables followed by a delay period of 1 s duration and an air-puff, in a quarter of trials.

On the first day, birds were allowed to discover the (window) perch and use it extensively to view the other bird. From the second day onward, a trial began when the experimenter sat continuously on the perch for 3.5 s (assessed via the Hall sensor signal), which triggered the playback of a stimulus. Stimuli were either S_1_ or S_10_ (the longest or shortest from each class), Fig. 1b,c. Our assumption was that S_1_ vs S_10_ are the most easily distinguishable stimuli from the set. One of the two stimuli was always associated with a negatively reinforcing air-puff delivered 2.4 s after trial onset (i.e, one second after the offset of the longest stimulus S_10_). On each trial, the played stimulus was pseudo-randomly selected; the puffed stimulus with probability 0.25 and the unpuffed stimulus with probability 0.75. Stimulus delivery was blocked after each trial for a random duration chosen uniformly in the interval from 12 to 15 s.

Note that in initial pre-training experiments in three birds, we observed that playing a puffed stimulus with probability 0.5 induced highly frequent escapes shortly after stimulus onset. By reducing the probability of the puffed stimulus to 0.25, we allowed birds to stay longer on the perch on average, thus producing a higher “surprise” by the aversively reinforcing air puff.

Over the course of the pre-training phase, we increased the duration of puffs from 0.03 s (light puff) to 1 s (strong puff). We roughly doubled puff duration at each increment. With increasing puff duration, the probability of displacing a bird from the perch increased. To avoid the puff, birds started to fly away from the perch to the other extreme of the cage (where there was no visibility of the neighboring cage).

Note that our task was designed to test birds’ sensory discrimination abilities with no requirement of motor learning. Because departing from a perch is part of the behavioral repertoire of all birds, we can rule out that superior motor skills in experimenters would have given them an advantage over observers during the testing phase.

*Training phase*

The training phase in experimenters began after the birds reached a performance criterion in the pre-training phase (see *Performance measures and Statistical Criterion*). In the training phase, all 10 stimuli were presented in pseudorandom order with the probability of a puffed stimulus P­­_puff_ = 0.25 and a puff duration of 1 s. The training phase ended at most a few days after the birds reached the criterion on all 10 stimuli.

*Pre-testing phase in observers and control groups*

After observers and birds from the PL and VL control groups were introduced into the experimenters’ cage, they were subjected to a *pre-testing* phase identical to the pre-training phase in experimenters. In the pre-testing phase, birds were exposed to stimuli S_1_ and S_10_ and ensuing air-puffs.

*Testing phase*

The testing phase in observers was identical to the training phase in experimenters (we refer to it as” testing” because we are testing whether the observer learnt from observation).

*Escape time bias*

To determine whether birds left the perch early or late in a trial we computed the escape time bias as the average deviation of escape time from the trial halftime. That is, the escape time bias is defined as

$$t_{bias}=\bar{t}_{esc}-\frac{T}{2}=\frac{1}{P(T)}\int_{0}^{T} tp(t)dt-\frac{T}{2},$$

where $T=2.4$ s is the duration of a trial, $\bar{t}_{esc}$ the average escape time, $P(T)$ the cumulative escape probability at trial end, and $p(t)$ the (instantaneous) escape probability at time $t.$ In practice, we binned time into 100 ms bins, which means that we replaced the integral with summation over 24 discrete time bins. We refer to as “escape time bias” because it reflects the extent to which an animal prefers to leave at a certain time in a trial compared to leaving uniformly throughout the trial. To ensure that$p(t)$was a true probability distribution, we computed escape time bias only on trials in which the bird escaped.

*Observer perching behavior*

In three pairs of EXP and OBS, we measured the perching behavior of the observer to test if observers tended to imitate the experimenter during the latter’s training phase. We computed Pearson correlation coefficients between the binary response vectors (escape/no escape) per trial of a each EXP and OBS pair (n= 3) for the entire training phase of the EXP (bird pair 1: r = 0.018, p = 0.09; pair 2: r = 0.12, p = 10^-52^; pair 3: r = -0.05, p = 10^-4^; p-values are small due to the large sample sizes (N = 9075, 15893, 6226 trials per pair, respectively)). Second, we looked at Pearson correlation coefficients between the binary response vectors only at the end of the training phase (last 1000 trials) in order to compare the performance of the expert EXP to its OBS (Bird pair 1: r = -0.01, p = 0.005; pair 2: r = -0.024, p = 0.44; pair 3: r = 0.024, p = 0.45). Therefore, we find no direct evidence that observational learning in the sense of action imitation plays an essential role in our social learning task.

*Robustness of statistics*

1. Computing dPesc values in blocks of 200 trials (instead of 100-trial blocks) and using the z-test (as mentioned in Methods, Performance measures and statistical criterion), we still obtained significant differences in trials to criterion between EXP and OBS (EXP: 4.02 ± 1.8*10^3^, OBS : 1.95 ± 0.67 ; p = 0.003, test statistic = 78, Wilcoxon rank sum test). Also, GENOBS still needed significantly more time to reach criterion than GENEXP (GENEXP: 1.8 ± 0.94*10^3^, GENOBS: 4.24 ± 3.4*10^3^, p = 0.023, test statistic = 64.5 Wilcoxon rank sum test).
2. When we changed the learning criterion from 7/8 to 4/4 blocks significant (again with z-test), we found our results unchanged: experimenters were still slower than observers (EXP: 3.71 ± 3.05*10^3^ trials, OBS: 1.27 ± 1.55*10^3^ trials; p = 0.013, test statistic = 72.5, Wilcoxon rank sum test), and GENOBS were still slower than GENEXP to reach criterion on the generalization set (GENEXP: 0.62 ± 0.48*10^3^ trials, GENOBS: 3.45 ± 3.17*10^3^trials; p = 0.007, test statistic = 71, Wilcoxon rank sum test).
3. Our results did not depend on hypothesis details. When we performed comparisons of group medians using two-tailed Wilcoxon tests instead of one-tailed tests, we found all results to be upheld (for example: trials to criterion EXP vs OBS: p = 0.015, Wilcoxon rank sum; trials to criterion PL vs OBS, p = 0.016; VL vs OBS, p = 0.003).
4. We explored the sensitivity of the trials to criterion on the birds’ probabilistic perching behavior. When we extracted true positive and false positive classification rates from the data (from EXP and OBS, average over first 300 trials in training/testing phase for each bird) and performed Monte-Carlo simulations (6000 trials, 50 experiments per bird), the criterion was reached earlier under the 3/4 blocks criterion compared to the 7/8 blocks criterion (3/4 case, average trials to criterion per bird: EXP 3.7 ± 2.3*10^3^, OBS 1.9 ± 2.4*10^3^ ; 7/8 case: EXP 5.3 ± 1.6*10^3^ , OBS 2.7 ± 2.5*10^3^). However, in either case, EXP were considerably slower than OBS.
5. We also performed Monte-Carlo simulations with true positive and false positive rates taken from later times in the training phase (average from 1200 to 1500 trials after training/testing phase onset). Again, EXP were considerably slower than OBS (3/4 case, average trials to criterion per bird: EXP 3.9 ± 2.4*10^3^, OBS 1.2 ± 1.8*10^3^; 7/8 case: EXP 5.0 ± 2.1*10^3^, OBS 2.1 ± 2.2*10^3^). Note that both EXP and OBS in these simulations took slightly longer than EXP and OBS in experiments (Fig. 2).
6. Here we report the mean and standard deviation for our behavioral measures (trials to criterion and dPesc) as well as Cohen’s d effect size for comparisons between two distributions in terms of the means. A) Trials to criterion for EXP: 4.8 ± 2.9*10^3^ (mean ± standard deviation) and OBS: 1.82 ± 1.7*10^3^. Cohen’s d effect size 1.22 for the difference in mean trials to criterion between EXP and OBS. B) dPesc at criterion for EXP: 0.36 ± 0.06 and OBS: 0.47 ± 0.11. Cohen’s d effect size 1.38 for the difference in dPesc at criterion between EXP and OBS. (C) Trials to criterion for GENEXP: 1.1 ± 0.5*10^3^ and GENOBS: 4.9 ± 4.0*10^3^, Cohen’s d = 1.3. (D) Average dPesc in the first three bins of the testing phase in GENEXP: 0.42 ± 0.08 and in GENOBS: 0.2 ± 0.18, Cohen’s d = 1.11. (D) Trials to criterion for PL: 6.32 ± 6.3*10^3^, Cohen’s d=1.04 for the difference in mean trials to criterion between OBS and PL (effect size = 1.29 without the outlier PL bird). (E) dPesc at criterion for PL: 0.32 ± 0.2. Cohen’s d=0.93 for the difference in mean dPesc at criterion between PL and OBS. (F) Trials to criterion for VL: 8.0 ± 2.5 *10^3^, Cohen’s d=3.11 for the difference in mean trials to criterion between VL and OBS. (F) dPesc at criterion for VL: 0.25 ± 0.11, Cohen’s d=1.87 for the difference in mean dPesc between VL and OBS. Cohen’s d=1.14 for the difference in mean dPesc between VL and EXP. (G) Trials to criterion for -TCOM: 1.74 ± 1.78*10^3^, Cohen’s d=0.05 for the difference in mean trials to criterion between -TCOM and OBS. (H) dPesc in the first 100 trials of the pre-training phase for EXP: 0.04 ± 0.08 and OBS: 0.09 ± 0.3. The mean and standard deviation of call frequencies in the stimulus period on puffed trials: 0.46 ± 0.26. The mean and standard deviation of call frequencies in the stimulus period on unpuffed trials: 0.39 ± 0.22. The mean and standard deviation of call frequencies in the delay period on puffed trials: 0.27 ± 0.15. The mean and standard deviation of call frequencies in the delay period on unpuffed trials: 0.42 ± 0.26.
7. After reaching the criterion, OBS and GENOBS discriminated the stimuli equally well (similar dPesc at criterion, OBS – GENOBS, difference in medians = 0.1, p = 0.077, test statistic = 61, two-sided Wilcoxon rank sum test, 95% CI = [-0.03, 0.31]).
8. On the generalization set, GENEXP were more accurate than GENOBS after reaching the criterion, but the difference was not significant (GENEXP: 0.53 ± 0.14, GENOBS: 0.34 ± 0.19, p = 0.129, test statistic = 36, Wilcoxon sign rank test).

**Observation learning was not a simple form of stimulus enhancement**

We did not find evidence that simple stimulus enhancement^16,17^ could account for observers’ rapid discrimination learning. Here, stimulus enhancement is defined as learning in an observing animal through the increased interaction with a particular stimulus after a demonstrating animal has directed its attention to the stimulus. Hoppitt and Laland^18^ provide a necessary condition for stimulus enhancement: observers must exhibit higher response rates to enhanced stimuli, which implies that enhancement could reveal itself as an increase in stimulus-contingent escape behavior even when there is no reinforcement.

To probe for stimulus enhancement, we quantified escape behavior during the first 100 pre-testing trials during which two auditory stimuli were paired with air-puffs that were too weak to displace a bird from the perch. During these trials, OBS exhibited a dPesc of 0.015 [-0.4, 0.77] that was not significantly different from zero (p = 0.44, test statistic = 24, 95% CI = [-0.17 0.44]; Wilcoxon signed rank test), Supplementary Fig. 3b. Hence, OBS were not initially drawn to either puffed or unpuffed stimuli. Rather, they expressed discriminative behavior only during later trials of the pre-testing phase, after we increased the strength of air-puffs. Thus, it seems that in addition to the stimuli and the actions of experimenters, observers needed also the aversive experience of air-puffs to express their learned knowledge.

**Note on dynamics of regularization parameter for L1-norm penalized logistic regression**

To achieve robust generalization, the value of λ had to grow at a rate $\alpha$ slower than the synaptic learning rate $\eta$of the logistic neuron ($\alpha\ll\eta$). In numerical simulations, we found that the value of λ converged to a positive value, Fig 5c. Training and generalization performance for the experimenter and observer were similar when λ was pre-fixed (to 0.013) or when it was dynamically altered.

**Effects of noisy experimenter actions on learning in observer neurons**

We were unable to assert from our simulations whether the apparent learning cue in observers was the air-puff’s auditory cue (e.g., the experimenter’s actions drew attention to the puff first, followed by a complex form of stimulus enhancement) or the experimenters’ escape behaviors (as in action imitation). That is, the simulation results matched well with experimental data both when the learning cues for the observer neuron were the air-puff sounds (Supplementary Fig. 5, solid lines) and when the cues were the modelled escape events (we modeled the escapes as binary random variables with a 30% chance of not representing the true class label, corresponding with the average false positive and false negative rates of EXP of about 30%, Supplementary Fig. 5, dashed lines). When we endowed the observer neuron with the same regularization constant λ as the experimenter neuron, but let the neuron learn not from presence/absence of air-puff sounds but from the experimenter’s noisy actions (see Methods), then training and generalization curves in the observer neuron were very similar and thus not representative of the data, (Supplementary Fig. 5, dot-dashed lines).

**Supplementary References**

1. Lohr, B. & Dooling, R. J. Detection of changes in timbre and harmonicity in complex sounds by zebra finches (Taeniopygia guttata) and budgerigars (Melopsittacus undulatus). *J. Comp. Psychol.* **112,** 36–47 (1998).

2. Cynx, J., Williams, H. & Nottebohm, F. Timbre discrimination in zebra finch (Taeniopygia guttata) song syllables. *J. Comp. Psychol.* **104,** 303–308 (1990).

3. Scharff, C., Nottebohm, F. & Cynx, J. Conspecific and heterospecific song discrimination in male zebra finches with lesions in the anterior forebrain pathway. *J. Neurobiol.* **36,** 81–90 (1998).

4. Woolley, S. C. & Doupe, A. J. Social context-induced song variation affects female behavior and gene expression. *PLoS Biol.* **6,** e62 (2008).

5. Sturdy, C. B., Phillmore, L. S., Price, J. L. & Weisman, R. G. Song-note discriminations in zebra finches (Taeniopygia guttata): Categories and pseudocategories. *Journal of Comparative Psychology* **113,** 204–212 (1999).

6. Lohr, B., Dooling, R. J. & Bartone, S. The discrimination of temporal fine structure in call-like harmonic sounds by birds. *J. Comp. Psychol.* **120,** 239–251 (2006).

7. Dooling, R. J. Temporal summation of pure tones in birds. *J Acoust Soc Am* **65,** 1058–1060 (1979).

8. Ohms, V. R., Gill, A., van Heijningen, C. A. A., Beckers, G. J. L. & ten Cate, C. Zebra finches exhibit speaker-independent phonetic perception of human speech. *Proc Biol Sci* **277,** 1003–1009 (2010).

9. Spierings, M. J. & ten Cate, C. Zebra finches are sensitive to prosodic features of human speech. *Proc. R. Soc. B Biol. Sci.* **281,** (2014).

10. Tchernichovski, O., Mitra, P. P., Lints, T. & Nottebohm, F. Dynamics of the vocal imitation process: how a zebra finch learns its song. *Science* **291,** 2564–9 (2001).

11. Derégnaucourt, S., Poirier, C., Kant, A. Van Der & Linden, A. Van Der. Comparisons of different methods to train a young zebra finch ( Taeniopygia guttata ) to learn a song. *J. Physiol. Paris* **107,** 210–218 (2013).

12. Canopoli, A., Herbst, J. & Hahnloser, R. H. R. A higher sensory brain region is involved in reversing reinforcement-induced vocal changes in a songbird. *J. Neurosci.* **34,** 7018–7026 (2014).

13. Tokarev, K. & Tchernichovski, O. *A novel paradigm for auditory discrimination training with social reinforcement in songbirds*. *bioRxiv* (Cold Spring Harbor Labs Journals, 2014).

14. Rescorla, R. & Wagner, R. A theory of Pavlovian conditioning: Variations in the effectiveness of reinforcement and nonreinforcement. *Class. Cond. II Curr. Res. Theory* **21,** 64–99 (1972).

15. Zentall, T. R. & Hogan, D. E. Concept Learning in the Pigeon : Transfer to New Matching and Nonmatching Stimuli. *Am. J. Psychol.* **88,** 233–244 (1975).

16. Zentall, T. R. Imitation: definitions, evidence, and mechanisms. *Anim. Cogn.* **9,** 335–53 (2006).

17. Byrne, R. W. Imitation as behaviour parsing. *Philos. Trans. R. Soc. Lond. B. Biol. Sci.* **358,** 529–36 (2003).

18. Hoppitt, W. & Laland, K. N. *Social learning: An introduction to mechanisms, methods, and models*. *Princeton University Press* (2013).
